# Towards quantitative accuracy in first-principles transport calculations: The GW method applied to alkane/gold junctions

**DOI:** 10.3762/bjnano.2.82

**Published:** 2011-11-09

**Authors:** Mikkel Strange, Kristian S Thygesen

**Affiliations:** 1 Center for Atomic-scale Materials Design, Department of Physics Technical University of Denmark, DK - 2800 Kgs. Lyngby, Denmark

**Keywords:** alkanes, density functional theory, electron transport, gold junction, GW

## Abstract

The calculation of the electronic conductance of nanoscale junctions from first principles is a long-standing problem in the field of charge transport. Here we demonstrate excellent agreement with experiments for the transport properties of the gold/alkanediamine benchmark system when electron–electron interactions are described by the many-body GW approximation. The conductance follows an exponential length dependence: *G_n_* = *G_c_* exp(−β*n*). The main difference from standard density functional theory (DFT) calculations is a significant reduction of the contact conductance, *G_c_*, due to an improved alignment of the molecular energy levels with the metal Fermi energy. The molecular orbitals involved in the tunneling process comprise states delocalized over the carbon backbone and states localized on the amine end groups. We find that dynamic screening effects renormalize the two types of states in qualitatively different ways when the molecule is inserted in the junction. Consequently, the GW transport results cannot be mimicked by DFT calculations employing a simple scissors operator.

## Introduction

The conductance of a molecule sandwiched between metallic electrodes is sensitive to the chemical and electronic structure of the molecule as well as the detailed atomic structure of the metal–molecule contact. Variations in the contact geometry beyond experimental control lead to an undesired spread in the measured conductance properties. For the most commonly used anchoring group, –thiol, these effects are rather pronounced due to the many possible contact geometries resulting from the strong Au–S interaction. Amine groups have been shown to produce more well-defined transport properties [1], which can be understood from the relatively weak Au–NH_2_ bond leading to larger structural selectivity [2].

Even for a given junction geometry, a quantitatively accurate description of electron transport from first principles remains a formidable task. Numerous studies based on density functional theory (DFT) have shown a significant overestimation of conductance relative to experimental values [3–14] (an exception to this trend occurs for small molecules, such as H_2_ [15–17] and CO [18–19], which chemisorb strongly to the electrodes, resulting in resonant transport through broad, partially filled resonances). The inability of DFT to describe off-resonant tunneling in the simplest molecular junctions limits the predictive power of the DFT-based approach to qualitative trends. It is now broadly accepted that the failure of DFT is mainly due to its incorrect description of the molecular energy levels. Indeed, physically motivated correction schemes have shown that much-improved agreement with experiments can be obtained after shifting the DFT molecular energy levels [13–14]. Such corrections are supposed to remove the self-interaction errors inherent in standard DFT exchange–correlation (xc) functionals [20–22] and account for image charge effects induced by the metal contacts. The drawback of the approach is that it assumes a weak coupling between molecular orbitals and metal states and treats the image-plane position as a free parameter.

The (self-consistent) GW approximation [23], which is rooted in many-body perturbation theory, was recently found to yield a considerable improvement over DFT for the conductance of gold/benzenediamine junctions [24]. Physically, the GW approximation corresponds to Hartree–Fock theory with the bare Coulomb interaction *v* = 1/|**r** − **r**′| replaced by a dynamically screened Coulomb interaction *W*(ω) = ε^−1^(ω)*v*. In contrast to standard DFT, the GW approximation is almost self-interaction free [25] and includes screening effects through the correlation part of the self-energy [26–28]. As a consequence, it provides quantitatively accurate predictions of energy gaps in systems with highly diverse screening properties, ranging from isolated molecules [29–30], through to semiconductors [31] and metals [32]. The broad applicability of the GW approach becomes particularly important for a metal–molecule interface where the electronic structure changes from insulating to metallic over a few angstroms.

In this work we use the GW approximation to study the role of exchange–correlation effects for the energy-level alignment and electron transport in short alkane chains coupled to gold electrodes through amine linker groups. The gold/alkane junction is a benchmark system for molecular charge transport and has been exhaustively investigated experimentally [1,12,33–44]. We focus here on the amine-linked alkanes to avoid the uncertainties related to the gold–thiol contact geometry, which is presently under debate [45–50]. We note that very recently it was shown that alkanes can be bound directly to gold electrodes without the use of anchoring groups [51].

The transport mechanism in (short) saturated molecular wires is coherent tunneling through molecular orbitals with energy far from the Fermi energy. The trend of conductance versus chain length (*n*) thus follows an exponential law of the form

[1]
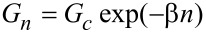


Recent experimentally reported values for the decay constant β of alkane-α,ω-diamine/gold junctions are in the range 0.9–1.0 per C atom [1,12,36], but earlier measurements also showed values around 0.8 [35]. Although previous studies based on DFT yielded β values within the experimental range, the contact conductance, *G_c_*, is typically overestimated by around an order of magnitude [3–10]. A study based on the many-body configuration interaction method has shown similar β values, but slightly reduced *G_c_* values, as compared to DFT [52]. By comparing DFT and GW calculations for C*_n_*-alkanediamine molecules with *n* = 2,4,6 we show that the erroneous *G_c_* values are a result of the incorrect level alignment in the DFT calculations. Indeed, GW yields a *G_c_* in close agreement with the experimental values. We find a pronounced orbital and length dependence of the quasiparticle (QP) corrections to the DFT energies, resulting from the different shape and localization of the molecular orbitals. The QP corrections range from −0.5 to −2.5 eV and can be qualitatively explained from a classical image-charge model.

## Method

**Figure 1:**
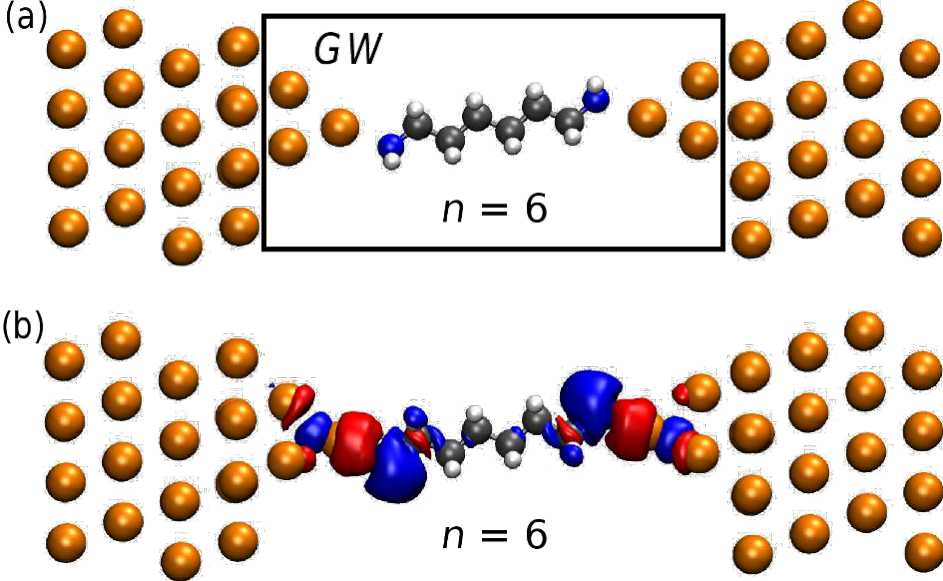
(a) Supercell used to model the gold/alkanediamine junctions. Similar supercells were used for *n* = 2 and *n* = 4 (not shown) and the key structural parameters can be found here [50]. The black box indicates the region of the extended molecule within which the GW self-energy is evaluated. (b) Isosurface of the electron-density difference between a DFT ground-state calculation and a constrained DFT calculation with one less electron on the molecule.

The junction geometries were optimized by means of the real-space projector-augmented wave method GPAW [53–54] with a grid spacing of 0.2 Å and the PBE functional for exchange and correlation (xc) [55]. The molecules were attached to Au(111) surfaces, modeled by an eight-layer-thick 4 × 4 slab, through small four-atom tips as shown in Figure 1a. The surface Brillouin zone was sampled on a 4 × 4 Monkhorst pack *k*-point grid, and the structures including molecule, Au tips, and outermost Au surface layers were relaxed until the residual force was below 0.03 eV/Å. We considered *n*-alkanediamine junctions with *n* = 2, 4 and 6. The key structural parameters can be found here [56]. For calculations of the molecules in the gas-phase, we include 16 Å of vacuum between molecules in the repeated supercells. All transport calculations where performed according to the method described in detail in [24]. In brief, we employ a basis set of numerical atomic orbitals corresponding to double-zeta plus polarization (DZP) for the Au atoms and double-zeta (DZ) for the atoms of the molecules. We use rather diffuse basis functions with a confinement-energy shift of 0.01 eV. This ensures that the calculated work function of Au(111) and the Kohn–Sham energy levels of the molecular junction are within 0.1 eV of those obtained from accurate grid calculations [24]. The transmission function is obtained from the Meir–Wingreen transmission formula [57–58]

[2]
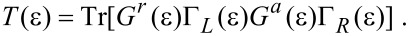


The retarded Green’s function of the extended molecule is calculated from

[3]
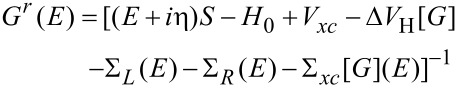


Here *S*, *H*_0_, and *V_xc_* are the overlap matrix, Kohn–Sham Hamiltonian and the local xc-potential in the atomic orbital basis, respectively; η is a positive infinitesimal.

The lead self-energy, Σ*_L_*_/_*_R_*, incorporates the coupling to the left and right electrodes and is obtained by standard techniques [59]. The term Δ*V_H_* gives the change in the Hartree potential relative to the DFT Hartree potential already contained in *H*_0_. Finally, the last term is the many-body xc self-energy, which in this work is either the bare exchange potential, *V_x_*, corresponding to Hartree–Fock, or the GW self-energy. As indicated both the Hartree potential and the xc self-energy depend on the Green’s function. The latter is evaluated fully self-consistently using a simple linear mixing of the Green functions. We represent all energy dependent quantities in Equation 3 on a large energy grid ranging from −200 eV to 200 eV with an energy-grid spacing of 0.01 eV.

The GW self-energy is evaluated for the extended molecule (indicated by the box in Figure 1a). However, only the part corresponding to the molecule is used while the remaining part is replaced by the DFT xc-potential. This is done to include nonlocal correlation (image-charge) effects from the electrodes in the GW self-energy of the molecule while preserving a consistent description of all metal atoms at the DFT level. We have verified that the calculations are converged with respect to the size of the extended molecules, see [24] for more details. The basis functions on the Au tip atoms extend a fair distance into the Au electrode and thus also describe screening effects to some extent in this region. The rather fast convergence of the screening effects with extended molecule size can be probed directly through a simple approach based on a constrained DFT calculation where the number of electrons on the molecule is constrained to be one less, or one more, than the number of electrons in a ground state DFT calculation. We measure the number of electrons on the molecule using a Mulliken charge analysis. The constrained DFT calculations are performed as self-consistent DFT calculations with the rigid shift of the molecular orbitals adjusted until the number of electrons is one less, thereby probing the highest occupied molecular orbital. We show in Figure 1b the isosurface of the electron-density difference obtained from a ground state DFT calculation and a constrained DFT calculation. The change in the electron density in the metal can be seen as the formation of an image charge (red color), which to a large extent is localized on the Au tip atoms closest to the molecule.

## Results and Discussion

### Energy-level alignment

The alignment of the molecular energy levels relative to the electrode Fermi level is of great importance for the transport properties of molecular junctions and seems to be the dominant effect at low bias voltage. At higher bias voltages, many-body calculations on small model systems suggest that electron correlations induce additional shifting and broadening of the molecular levels, which can also affect the transport properties [60]. Here we focus on the low-bias regime and postpone consideration of the finite-bias effects to a later study.

**Figure 2:**
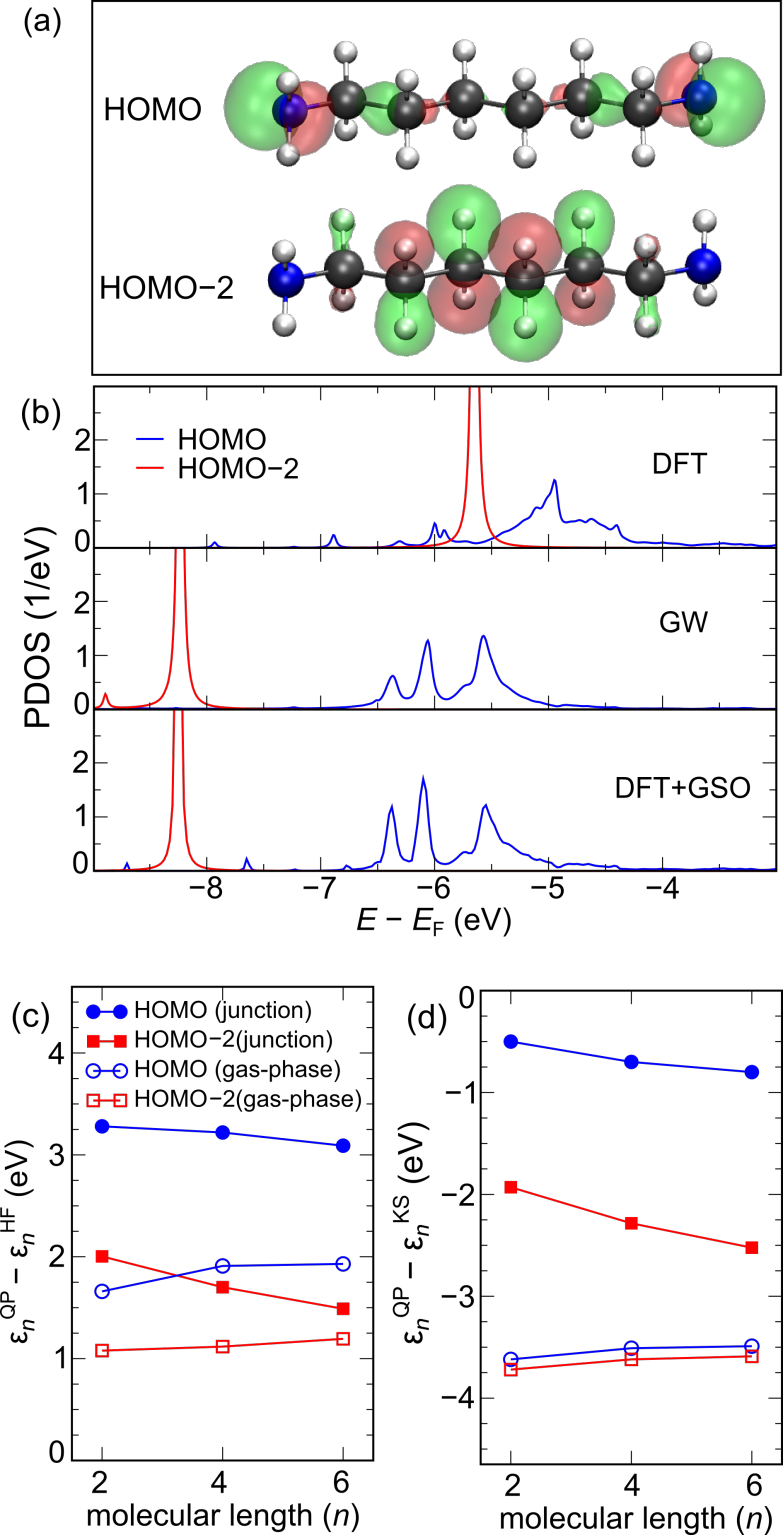
(a) Isosurfaces for the HOMO and HOMO−2 orbitals of the C_6_-alkanediamine molecule. (b) HOMO and HOMO−2 PDOS in the junction obtained from DFT-PBE (top), GW (middle) and DFT with a generalized scissors operator (bottom). (c) Quasiparticle corrections to the Hartree–Fock levels in the gas-phase (open symbols) and in the junction (filled symbols) as a function of molecular length *n*. The HOMO and HOMO−2 are denoted by circles and squares, respectively. (d) Same as (c) but for DFT rather than Hartree–Fock.

The molecular orbitals (MOs) of the alkanediamine chains comprise states that are delocalized over the carbon backbone and states that are localized on the NH_2_ end group. We shall consider the highest occupied molecular orbital (HOMO) and HOMO–2 as representatives for the two classes of states (Figure 2a). We note that the HOMO–1 is similar to the HOMO, with slightly lower energy given by the coupling of the two end groups across the wire. In Table 1 and Table 2 we list the energy of the HOMO and HOMO–2 calculated with DFT-PBE, Hartree–Fock (HF), and GW for the molecules in the gas-phase and in the junction.

**Table 1:** Calculated HOMO and HOMO–2 energies aligned to the vacuum level and in units of eV.

method	orbital	*n* = 2	*n* = 4	*n* = 6

DFT-PBE	HOMO	−4.9	−5.1	−5.1
	HOMO–2	−8.5	−8.2	−8.0
HF	HOMO	−10.2	−10.5	−10.5
	HOMO–2	−13.3	−12.9	−12.8
GW	HOMO	−8.5	−8.6	−8.6
	HOMO–2	−12.2	−11.8	−11.6

**Table 2:** Calculated HOMO and HOMO–2 energies in the junction relative to the electrode Fermi level.

method	orbital	*n* = 2	*n* = 4	*n* = 6

DFT-PBE	HOMO	−4.3	−4.2	−4.4
	HOMO−2	−6.5	−5.9	−5.7
HF	HOMO	−8.1	−8.1	−8.3
	HOMO–2	−10.4	−9.9	−9.7
GW	HOMO	−4.8	−4.9	−5.2
	HOMO–2	−8.4	−8.2	−8.2

In the gas-phase, all three methods predict the HOMO energy to be almost independent of molecular length. This is clearly due to its end-group-localized character. In contrast, the energy of the HOMO–2 level shifts upward in energy as the molecular length increases. This reflects its extended nature and can be interpreted as a band-discretization effect. To the best of our knowledge no experimental results exist for the ionization potential of alkanediamine molecules. However, for the closely related butane molecule (C_2_ alkane with CH_3_ end groups) we obtain a GW-calculated HOMO energy of −11.4 eV in very good agreement with the experimental ionization potential of 11.2 eV [61]. In comparison, the DFT-PBE HOMO energy is severely underestimated at −7.9 eV. This finding agrees well with previous studies on a broader range of small molecules [24,29–30].

In the junction, the molecular orbitals, 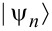, were obtained by diagonalizing the DFT Hamiltonian corresponding to the molecule. The projected density of states (PDOS) of such a state is then given by the spectral function, 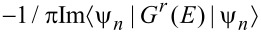, where *G* is the appropriate Green’s function (calculated with DFT, HF, or GW). The level position is defined as the first moment of the PDOS. Figure 2b shows the PDOS for the HOMO and HOMO–2 for the C_6_-alkanediamine junction as calculated with DFT-PBE (upper panel) and GW (middle panel). The lower panel shows the PDOS obtained from a DFT calculation where the molecular levels have been shifted to match the GW levels, i.e., after adding to the Kohn–Sham Hamiltonian a generalized scissors operator of the form


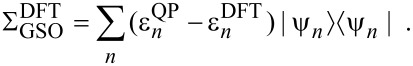


Here, the 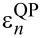 denote the QP energy obtained from the GW calculation. We see that the main features of the GW spectral function can be well reproduced by the shifted DFT Hamiltonian, although small differences remain. A similar conclusion was reached in [24] for a gold/benzenediamine junction.

The molecular orbital energies from a GW calculation include the dynamical response of the electron system to an added or removed electron through the correlation part of the self-energy. In general, correlations tend to shift the filled levels upwards and the empty levels downwards relative to the bare Hartree–Fock energies. This is because the inclusion of screening reduces the energy cost of removing/adding electrons to the molecule. When a molecule is brought into contact with a metallic junction its environment changes from insulating to metallic. This implies extra screening of an added or removed electron, which will cause the filled levels to shift upwards and the empty levels to shift downwards even more than for the isolated molecules, i.e., the gap will shrink relative to its gas-phase value. It has been shown previously that DFT in (semi)local approximations and Hartree–Fock completely miss this important effect [26–28].

In Figure 2c and Figure 2d, we show the QP corrections to the HF and DFT Kohn–Sham energy levels as a function of molecular length. The results for the HOMO and the HOMO–2 are denoted by circles and squares. We notice first that the QP corrections are very significant with absolute values reaching almost 4 eV and with a pronounced orbital and length dependence. The Hartree–Fock QP corrections are all positive showing that HF places the occupied levels lower than predicted by GW. This is in contrast to the corrections to the DFT levels, which are all negative, in agreement with the well-known underestimation of ionization potentials as predicted from the negative Kohn–Sham HOMO energy obtained using LDA or GGA functionals. In contrast to Hartree–Fock the Kohn–Sham QP corrections are smaller for molecules in the junction compared to the gas-phase. In fact, the position of the HOMO level by DFT is relatively close the GW level position and only lies 0.5–0.8 eV higher. The fact that the DFT-PBE description of molecular energy levels is much better in the junction than in the gas-phase agrees with previous findings [24,27,62] and can be explained from the origin of the PBE functional in the homogeneous electron gas [63].

It is instructive to consider the shift in the molecular energy levels due to correlation effects coming from the metal electrodes. In simple terms this corresponds to the shift induced by image-charge effects. In order to isolate the part of the correlation energy originating from the metallic electrodes we define the quantity

**Figure 3:**
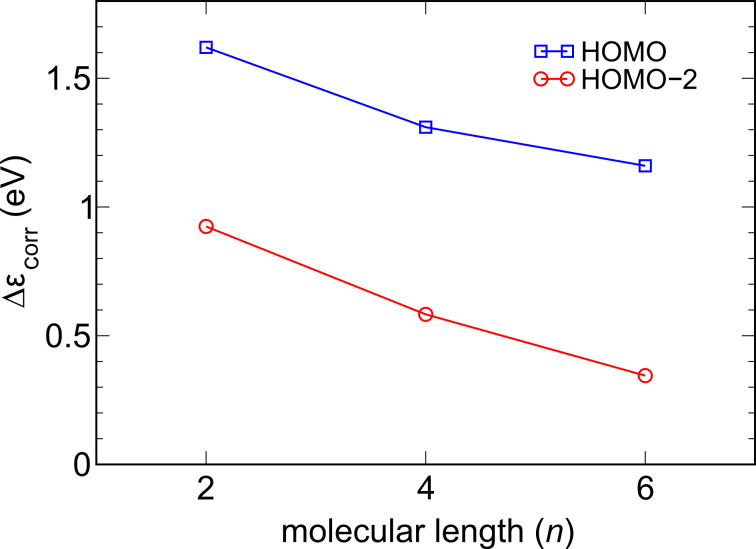
Change in the correlation energy of the HOMO and HOMO–2 energy levels when the molecules are taken from the gas-phase into the junction. This value represents the shift of molecular levels due to the enhanced screening provided by the metallic electrodes (an image-charge effect in simple terms).

[4]
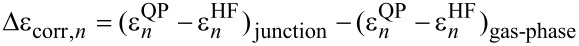


which is shown in Figure 3.

The result can be understood qualitatively by considering a classical model to account for the screening effect of the electrodes. Classically a charge distribution close to any surface will experience an image potential. The strength of the image potential in general depends on the dielectric constant of the surface material and the local geometrical shape of the surface. Here we model the Au electrodes as perfect metals. The image potential for a point charge halfway between two metal surfaces separated by a distance *L* is ≈ 10.0/*L* (eV·Å) [64]. This predicts that the image-charge effect is proportional to 1/*L*. The Au tip atoms in our simulations are about 8 Å apart for the C_2_-alkanediamine junction giving a rough estimate of 1.3 eV for the image-charge effect in qualitative agreement with the GW calculations. The HOMO experiences a larger image-charge effect than the HOMO–2, which can be understood from the fact that its charge density is located closer to the metallic surfaces. In the limit of an infinitely long wire the HOMO–2 will be spread out over the entire molecule and the image-charge effect should vanish. On the other hand, in this limit the HOMO would stay localized near the surface and therefore approach a nonzero constant image charge potential. If we model the HOMO charge density as a point charge of half an electron on each of the amine groups we can estimate this limiting constant to be 3.6/(2*d*) (eV·Å), where *d* is the distance to the nearest metal surface. Taking *d* to be about the same length as the Au–N bond (2.34 Å) gives a limiting value estimate of 0.8 eV. Again, this seems to be in qualitative agreement with our GW findings.

**Figure 4:**
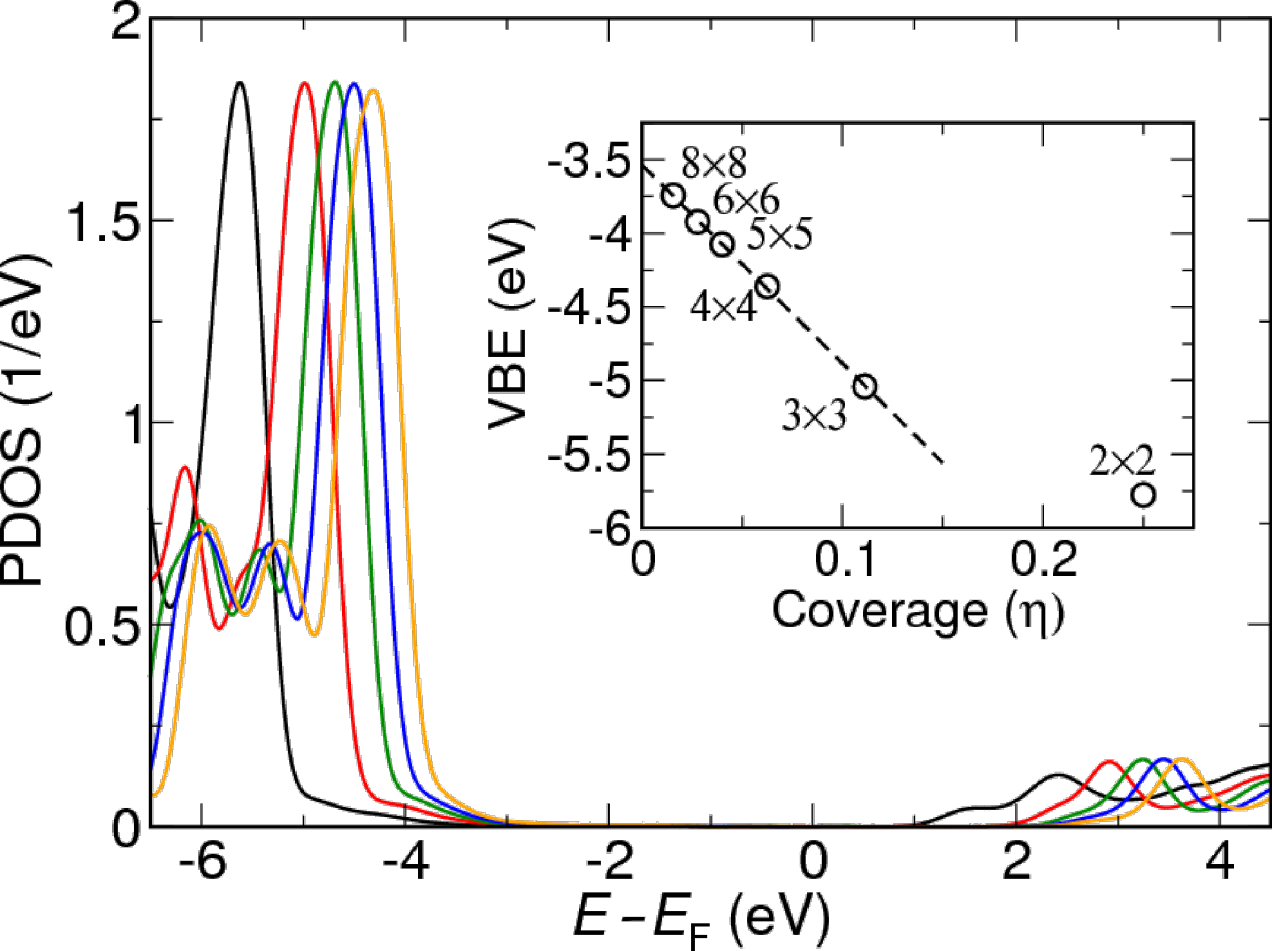
The molecular valence-band edge (or HOMO) as a function of coverage for an *n* = 6 alkane. The numbers indicate the number of surface atoms.

Finally, we discuss the coverage dependence of the energy level position for alkanediamine–Au junctions. It was shown in [65] that the DFT level position for amine-linked molecules is strongly dependent on the coverage. In contrast to the screening (image-charge) effects as discussed above, which appear in the correlation part of the self-energy, this is a purely electrostatic effect resulting from the localized surface dipoles formed at the Au–NH_2_ bond. To investigate the dependence of the energy levels on the coverage for our junctions, we have performed DFT calculations for a range of transverse supercell dimensions for the geometry shown in Figure 1. In Figure 4 we show the PDOS of a methylene unit in the central part of the molecule for transverse supercells with 2 × 2 up to 8 × 8 surface atoms.

The PDOS peaks and band edge shift up in energy as the transverse supercell size is increased, in agreement with the results of [65]. The inset shows the energy shift obtained from reading off the shift in the PDOS as a function of coverage defined as η = 1/*N*_surface_, where *N*_surface_ is the number of surface atoms. For a supercell of size 3 × 3 and larger, the shift is seen to be directly proportional to the coverage, as expected for a two-dimensional array of dipoles [66]. This allow us to extract the electrostatic shift corresponding to the single-molecule limit. We find that the electrostatic energy shift when going from a 4 × 4 supercell to the single-molecule limit is indeed significant with a value around 1 eV.

### Transport calculations

The transmission functions of the C_2_-, C_4_- and C_6_-alkanediamine junction geometries were calculated by using the GW and the PBE xc potential as approximations for Σ_xc_ in Equation 3. To include the coverage dependence, we simulated the low-coverage limit η = 0^+^ by performing calculations for the 4 × 4 junction (corresponding to η = 1/16) with all molecular levels shifted up by 1 eV through a simple scissors-operator self-energy.

**Figure 5:**
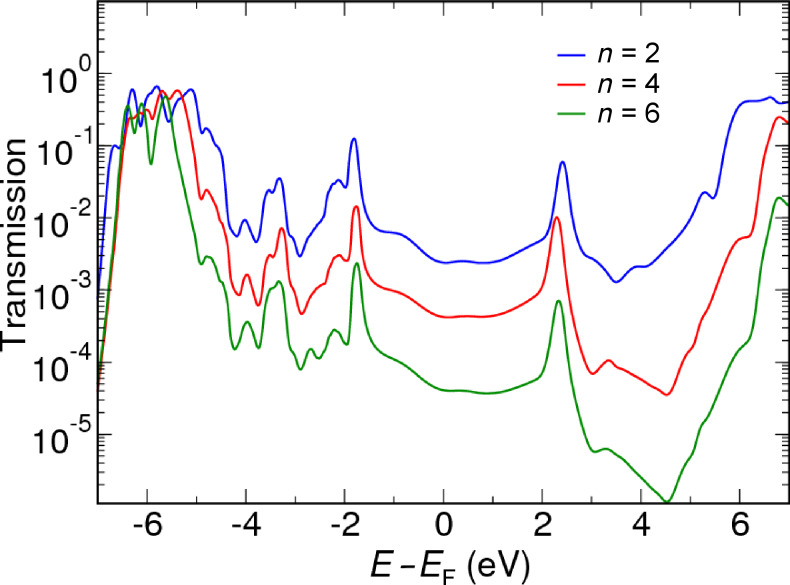
The transmission function calculated by GW for a molecular length of *n* = 2, *n* = 4 and *n* = 6 at a coverage of η = 1/16.

The transmission function calculated by GW for a coverage of η = 1/16 is shown in Figure 5 on a logarithmic scale. The transmission functions for different molecular lengths have very similar shapes in the important region near the Fermi level *E*_F_, however, the magnitude is increasingly suppressed as a function of the molecular length. The similarity of the transmission functions may at first seem surprising since we have shown that the position of the molecular energy levels shows some length dependence. In particular the HOMO level was found to decrease in energy by 0.5 eV when *n* increases from 2 to 6 (Table 2). This shift is indeed visible in the transmission function in the range −4.0 to −6.0 eV where the HOMO is located. On the other hand the features in the transmission function around the Fermi level are determined by the local electronic structure of the Au tips.

**Figure 6:**
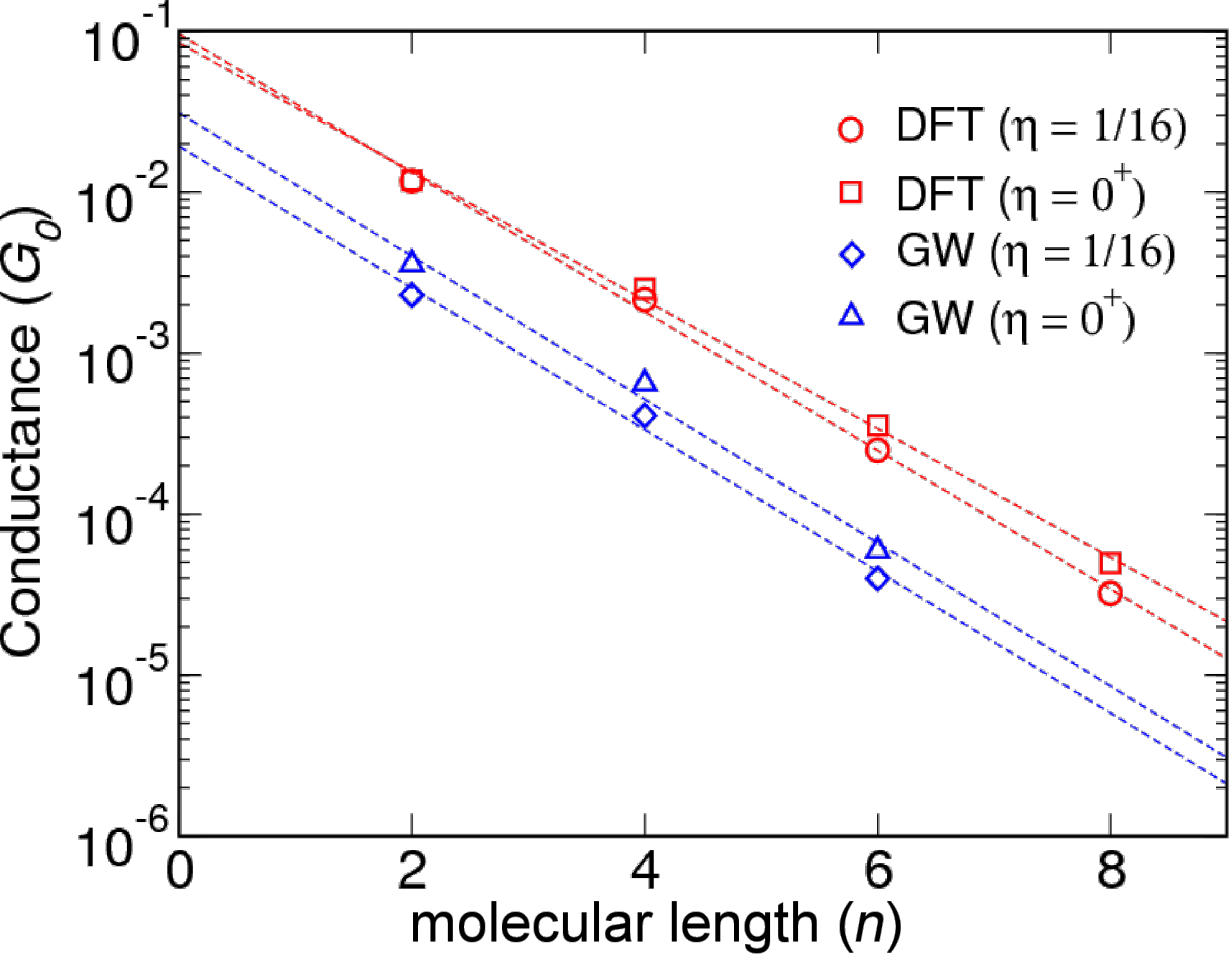
Calculated conductance plotted as a function of the molecular length for a coverage corresponding to 4 × 4 Au atoms per molecule (η = 1/16) and extrapolated to the single-molecule limit (η = 0).

The zero-bias conductance is obtained from the transmission function at the Fermi level, *G* = *G*_0_*T*(*E*_F_) where *G*_0_ = 2*e*^2^/*h* is the unit of quantum conductance. The zero-bias conductance is plotted in Figure 6 as a function of the molecular length. We have also included the DFT results for comparison. The dashed lines show the best fits to the exponential form *G_n_* = *G_c_*exp(−β*n*).

The values for β and *G_c_* corresponding to the single-molecule limit (η = 0) and 4 × 4 Au atoms per molecule (η = 1/16) are reported in Table 3 together with the experimental values. We note that the contact conductance was not stated in [12], but has been estimated by extrapolation to zero molecular length from the reported experimental data. When comparing to experiment it should be kept in mind that experiments are often performed in solution at room temperature and are subject to variations in the detailed atomic structure. However, it has been shown that amine-linked molecules bind preferentially to under-coordinated Au atoms, such as in the structures considered here, and show a relatively narrow conductance distribution [1].

**Table 3:** Calculated contact conductance *G_c_* in units of *G*_0_ and exponential decay constant β per carbon atom; η denotes the coverage.

	DFT		GW		exp.
η	1/16	0	1/16	0	

*G_c_*	0.11	0.10	0.02	0.04	0.02^a^, 0.030^c^
β	0.99	0.92	1.01	1.02	0.97^a^, 0.93^b^, 0.91^c^

^a^ [12] (*G_c_* is not stated, but is estimated from the reported data); ^b^ [31]; ^c^ [1].

The rather weak effect of coverage on the conduction properties is in agreement with the findings reported in [67] where a C_4_-alkanediamine/Au junction with 3 × 3 and 4 × 4 surfaces was considered. Our DFT results are in reasonable agreement with previous DFT studies showing decay factors in the range 0.83–1.01 and conductance resistances in the range (0.09–0.28) *G*_0_ [8–10]. While the β values obtained with GW are rather close to the DFT calculated ones, the contact conductance is reduced by a factor of 3–5, depending on the coverage. This is a direct result of the molecular levels lying further away from *E*_F_ (by 0.5–2.5 eV, Figure 2d) in GW compared to DFT.

## Conclusion

We have unraveled the important role of exchange–correlation effects for the energy-level alignment and low-bias conductance of gold/alkanediamine molecular junctions. Based on many-body GW calculations we found that the origin of the overestimation of the contact conductance, *G_c_*, by standard DFT is due to the incorrect energy-level alignment in the junction. The absence of self-interaction and the inclusion of image-charge screening effect through the GW self-energy improves the description of the energy levels and yields values for *G_c_* and the decay constant β that are in good agreement with experimental results. The quasiparticle corrections to the DFT energy levels showed a significant orbital dependence ranging from −0.5 eV to −2.5 eV due to the different shape and localization of the molecular orbitals. Our results demonstrate that quantitatively accurate calculations of conductance from first-principles are feasible, although computationally demanding.

## References

[1] Venkataraman L, Klare J E, Tam I W, Nuckolls C, Hybertsen M S, Steigerwald M L (2006). Nano Lett.

[2] Kristensen I S, Mowbray D J, Thygesen K S, Jacobsen K W (2008). J Phys: Condens Matter.

[3] Tomfohr J K, Sankey O F (2002). Phys Rev B.

[4] Sen A, Kaun C-C (2010). ACS Nano.

[5] Kaun C-C, Guo H (2003). Nano Lett.

[6] Müller K-H (2006). Phys Rev B.

[7] Paulsson M, Krag C, Frederiksen T, Brandbyge M (2009). Nano Lett.

[8] Wohlthat S, Pauly F, Reimers J R (2008). Chem Phys Lett.

[9] McDermott S, George C B, Fagas G, Greer J C, Ratner M A (2009). J Phys Chem C.

[10] Sheng W, Li Z Y, Ning Z Y, Zhang Z H, Yang Z Q, Guo H (2009). J Chem Phys.

[11] Li C, Pobelov I, Wandlowski T, Bagrets A, Arnold A, Evers F (2008). J Am Chem Soc.

[12] Hybertsen M S, Venkataraman L, Klare J E, Whalley A C, Steigerwald M L, Nuckolls C (2008). J Phys: Condens Matter.

[13] Quek S Y, Venkataraman L, Choi H J, Louie S G, Hybertsen M S, Neaton J B (2007). Nano Lett.

[14] Mowbray D J, Jones G, Thygesen K S (2008). J Chem Phys.

[15] Smit R H M, Noat Y, Untiedt C, Lang N D, van Hemert M C, van Ruitenbeek J M (2002). Nature (London).

[16] Djukic D, Thygesen K S, Untiedt C, Smit R H M, Jacobsen K W, van Ruitenbeek J M (2005). Phys Rev B.

[17] Garciá Y, Palacios J J, SanFabián E, Vergés J A, Pérez-Jiménez A J, Louis E (2004). Phys Rev B.

[18] Untiedt C, Dekker D M T, Djukic D, van Ruitenbeek J M (2004). Phys Rev B.

[19] Strange M, Thygesen K S, Jacobsen K W (2006). Phys Rev B.

[20] Toher C, Filippetti A, Sanvito S, Burke K (2005). Phys Rev Lett.

[21] Toher C, Sanvito S (2007). Phys Rev Lett.

[22] Ke S-H, Baranger H U, Yang W (2007). J Chem Phys.

[23] Hedin L (1965). Phys Rev.

[24] Strange M, Rostgaard C, Häkkinen H, Thygesen K S (2011). Phys Rev B.

[25] Nelson W, Bokes P, Rinke P, Godby R W (2007). Phys Rev A.

[26] Neaton J B, Hybertsen M S, Louie S G (2006). Phys Rev Lett.

[27] Garcia-Lastra J M, Rostgaard C, Rubio A, Thygesen K S (2009). Phys Rev B.

[28] Thygesen K S, Rubio A (2009). Phys Rev Lett.

[29] Rostgaard C, Jacobsen K W, Thygesen K S (2010). Phys Rev B.

[30] Blase X, Attaccalite C, Olevano V (2011). Phys Rev B.

[31] Hybertsen M S, Louie S G (1986). Phys Rev B.

[32] Holm B, von Barth U (1998). Phys Rev B.

[33] Xu B, Tao N J (2003). Science.

[34] Engelkes V B, Beebe J M, Frisbie C D (2004). J Am Chem Soc.

[35] Chen F, Li X, Hihath J, Huang Z, Tao N (2006). J Am Chem Soc.

[36] Park Y S, Whalley A C, Kamenetska M, Steigerwald M L, Hybertsen M S, Nuckolls C, Venkataraman L (2007). J Am Chem Soc.

[37] Huang Z, Chen F, Bennett P A, Tao N (2007). J Am Chem Soc.

[38] Widawsky J R, Kamenetska M, Klare J, Nuckolls C, Steigerwald M L, Hybertsen M S, Venkataraman L (2009). Nanotechnology.

[39] Kamenetska M, Koentopp M, Whalley A C, Park Y S, Steigerwald M L, Nuckolls C, Hybertsen M S, Venkataraman L (2009). Phys Rev Lett.

[40] Song H, Lee T, Choi N-J, Lee H (2007). Appl Phys Lett.

[41] Kim Y, Hellmuth T J, Buerkle M, Pauly F, Scheer E (2011). ACS Nano.

[42] Martin C A, Ding D, van der Zant H S J, van Ruitenbeek J M (2008). New J Phys.

[43] Nichols R J, Haiss W, Higgins S J, Leary E, Martin S, Bethell D (2010). Phys Chem Chem Phys.

[44] Haiss W, Martin S, Scullion L E, Bouffier L, Higgins S J, Nichols R J (2009). Phys Chem Chem Phys.

[45] Cossaro A, Mazzarello R, Rousseau R, Casalis L, Verdini A, Kohlmeyer A, Floreano L, Scandolo S, Morgante A, Klein M L (2008). Science.

[46] Wang Y, Chi Q, Hush N S, Reimers J R, Zhang J, Ulstrup J (2009). J Phys Chem C.

[47] Voznyy O, Dubowski J J, Yates J T, Maksymovych P (2009). J Am Chem Soc.

[48] Jadzinsky P D, Calero G, Ackerson C J, Bushnell D A, Kornberg R D (2007). Science.

[49] Walter M, Akola J, Lopez-Acevedo O, Jadzinsky P D, Calero G, Ackerson C J, Whetten R L, Grönbeck H, Häkkinen H (2008). Proc Natl Acad Sci U S A.

[50] Strange M, Lopez-Acevedo O, Häkkinen H (2010). J Phys Chem Lett.

[51] Cheng Z L, Skouta R, Vazquez H, Widawsky J R, Schneebeli S, Chen W, Hybertsen M S, Breslow R, Venkataraman L (2011). Nat Nanotechnol.

[52] Fagas G, Greer J C (2007). Nanotechnology.

[53] Enkovaara J, Rostgaard C, Mortensen J J, Chen J, Dulak M, Ferrighi L, Gavnholt J, Glinsvad C, Haikola V, Hansen H A (2010). J Phys: Condens Matter.

[54] Larsen A H, Vanin M, Mortensen J J, Thygesen K S, Jacobsen K W (2009). Phys Rev B.

[55] Perdew J, Burke K, Ernzerhof M (1996). Phys Rev Lett.

[R56] 56We use the equilibrium PBE lattice constant of 4.18 Å for Au. The distance between the second outermost Au(111) atomic surface layers in the left and right electrode was fixed at 21.59 Å, 24.10 Å and 26.63 Å for the *n* = 2, 4 and 6 junction, respectively. The resulting relaxed N–Au bond length are 2.34 Å, 2.35 Å and 2.33 Å.

[57] Meir Y, Wingreen N S (1992). Phys Rev Lett.

[58] Thygesen K S (2006). Phys Rev B.

[59] Thygesen K S, Jacobsen K W (2005). Chem Phys.

[60] Thygesen K S (2008). Phys Rev Lett.

[61] (2011). http://webbook.nist.gov/cgi/cbook.cgi?ID=C106978&Units=SI&Mask=20#Ion-Energetics.

[62] Dell’Angela M, Kladnik G, Cossaro A, Verdini A, Kamenetska M, Tamblyn I, Quek S Y, Neaton J B, Cvetko D, Morgante A (2010). Nano Lett.

[63] Rohlfing M (2010). Phys Rev B.

[64] Chen C J (1993). Introduction to Scanning Microscopy.

[65] Wang J-g, Prodan E, Car R, Selloni A (2008). Phys Rev B.

[66] Natan A, Kronik L, Haick H, Tung R T (2007). Adv Mater.

[67] Feng X Y, Li Z, Yang J (2009). J Phys Chem C.

